# Exploring gender disparities in academic orthopaedic surgery faculty: analyzing subspecialty and leadership diversity to foster inclusivity

**DOI:** 10.1186/s13018-025-06048-9

**Published:** 2025-07-11

**Authors:** Malini Anand, Kaitlyn R. Julian, Mary K. Mulcahey, Stephanie E. Wong

**Affiliations:** 1 Department of Orthopaedic Surgery, University of California, Davis, Sacramento, CA USA; 2 School of Medicine, University of California, San Francisco, San Francisco, CA USA; 3 Department of Orthopaedic Surgery and Rehabilitation, Loyola University Medical Center, Maywood, IL USA; 4 Department of Orthopaedic Surgery, University of California, San Francisco, San Francisco, CA USA

**Keywords:** Gender diversity, Inclusion, Faculty, Mentorship, Awareness, Equity

## Abstract

**Background:**

The historic gap in gender diversity within orthopaedic surgery is widely acknowledged and continues to persist. The lack of female representation in orthopaedic surgery has been attributed to a variety of factors, including the absence of female mentors and leaders within the field. As such, we sought to examine the gender diversity among orthopaedic surgery faculty in various subspecialties at academic institutions and the distribution of female faculty in positions of leadership.

**Methods:**

The American Medical Association Fellowship and Residency Electronic Interactive Database (FREIDA) was used to identify all allopathic orthopaedic surgery residency programs during the 2022 to 2023 academic year. The total number of faculty, and distribution of female faculty by subspecialty were collected from January to March 2023. The mean and percentage of female faculty in each subspecialty per program was calculated.

**Results:**

The total number of orthopaedic surgery female faculty identified was 524. The subspecialty with the highest percentage of female faculty per program was pediatrics at 26.1% (148/511). Hand (18.6%; 113/511), oncology (19.2%; 38/511), foot and ankle (13.6%; 49/511), spine (3.9%; 21/511), shoulder and elbow (7.4%; 7/511) and adult reconstruction (3.7%; 24/511) had lower percentages of female faculty per program. A total of 52 (10.2%) female section chiefs were identified across all programs. Oncology had the highest percentage of female faculty represented in section leadership at 18.4% (7) and sports medicine had the lowest at 4.8% (4).

**Conclusion:**

Gender diversity of faculty in orthopaedic surgery is low with adult reconstruction (3.7%), spine (3.9%), and shoulder and elbow (7.4%) having the lowest percentages of female faculty. The percentage of female faculty represented in section leadership is also lacking with a total of 52 (10.2%) female section chiefs identified across all programs. Increasing the number of females in leadership positions across all orthopaedic subspecialties may be one step in helping improve gender diversity in the field.

## Introduction

The percentage of female orthopaedic surgeons is among the lowest across all fields of medicine [4]. As of 2020, the percentage of female orthopaedic surgeons actively practicing in the United States was 6%, and the proportion of female orthopaedic residents in allopathic programs in the United States was 19% during the 2021–2022 academic year [7]. These numbers continue to remain low even as 53% of medical students enrolled in 2022 identified as female [16].

Furthermore, within the field of orthopaedic surgery, there has been a shift towards a greater number of surgeons seeking fellowship training opportunities following residency. A study by Horst et al. demonstrated that from 2003 to 2013, the percentage of orthopaedic surgeons pursuing fellowship gradually increased from 76 to 90% by 2013 [6]. As of 2023, 7.3% of orthopaedic surgery residents planned to pursue more than one fellowship [1]. Due to this trend in continually increasing sub-specialization and a higher number of applicants to fellowship programs, the number of positions available for subspecialty training has adjusted for demand. From 2010 to 2017, the number of fellowship positions expanded across all subspecialties, with the exception of spine and trauma [16]. Notably, the most substantial increase in the number of available positions occurred in adult reconstruction.

Female orthopaedic surgery residents comprise between 7 − 10% of all fellowship applicants [3]. Existing literature attributes the underrepresentation of females in orthopaedic surgery to a variety of factors, including the absence of female role models, and lack of exposure to orthopaedic surgery in medical school rotations [2]. Notably, Bratescu et al. conducted a survey of female orthopaedic surgery residents to investigate the factors influencing their choice of specific specialties [2]. The authors found that interest in the field, early exposure, and mentorship from female faculty were the most important factors. With the increasing specialization in orthopaedic surgery, it is necessary to assess the diversity within each of the subspecialties and analyze the factors contributing to gender disparities.

The questions and purpose of this study were to understand (1) the gender diversity among orthopaedic surgery faculty in various subspecialties at academic institutions and (2) the current distribution of female faculty in positions of leadership within each orthopaedic subspecialty at academic institutions.

## Methods

The American Medical Association (AMA) Fellowship and Residency Electronic Interactive Database was used to identify all allopathic orthopaedic surgery residency programs during the 2022–2023 academic year. All data for this study were collected from programs and hospital websites along with associated social media pages. Gender in this study was determined based on pronouns listed on the website, and names and photos when not available. Each hospital website was reviewed by two members of the research team and data correlated for accuracy. For programs without clear listings of position and gender online, program coordinators emailed for clarification and detailed demographic information. This information was used to verify or complete incomplete data extracted from program websites.

This study is a review of pre-existing available public data. As such no informed consent was required or obtained for this article and no identifying information of individuals is included.

While in this study we are studying gender, which is a variable social construct (WHO definition), and not sex, the terms female and male are used throughout this manuscript as these are the terms used in the majority previously of published works.

The total number of faculty, faculty based on gender, and the distribution of faculty by subspecialty, academic position, and section chief were collected from January 2023 to March 2023. The subspecialties that were evaluated were hand, sports medicine, trauma, foot and ankle, spine, trauma, pediatrics, shoulder and elbow, oncology, and adult reconstruction. Academic positions that were evaluated were those who were identified as “full professor” or “professor.” Assistant, associate, and emeritus professors were not included as full professors. In this study we focused on evaluating the position of full professor as criteria for assistant and associate professorship can vary significantly between institutions.

The data was collected and statistics were evaluated using Excel 2016. Percent faculty by gender was calculated out of the total number of faculty in each subspecialty.

Distributions of section chiefs and full professors were calculated out of the total male or female faculty in a specific subspecialty. The percentage of male faculty and female faculty by subspecialty in positions of section chief or full professor were then compared using chi square analysis. P Values less than 0.05 were considered statistically significant.

## Results

The total number of orthopaedic surgery female faculty identified was 524 (12.1%), out of a total of 4,303 faculty nationally associated with allopathic residency programs in the United States. Out of 153 orthopaedic surgery residency programs, 144 programs were included in the study, due to lack of publicly accessible information from nine programs. The distribution of faculty by gender and subspecialty is shown in Table 1.

**Table 1:** Distribution of female orthopaedic surgery faculty by Sub-specialty

	Total Faculty	Total Male Faculty	Percent Male Faculty	Total Female Faculty	Percent Female Faculty
Hand	606	493	81.3%	113	18.6%
Spine	530	509	96.1%	21	3.9%
Sports Medicine	805	722	89.6%	83	10.3%
Trauma	467	421	90.2%	46	9.8%
Foot and Ankle	294	254	86.4%	40	13.6%
Pediatrics	565	417	73.8%	148	26.1%
Shoulder and Elbow	147	136	92.5%	11	7.4%
Oncology	198	160	80.1%	38	19.2%
Adult Reconstruction	648	624	96.3%	24	3.7%

Pediatrics had the highest number of female faculty (148; 26.1%). The total number of female adult reconstruction faculty was 24, with an average of 3.7% of orthopaedic surgery adult reconstruction faculty at any given program being female. Adult reconstruction was found to have the least gender diversity of all orthopaedic subspecialty faculty.

A total of 52 (10.2%) female section chiefs and 808 (21.6%) male section chiefs were identified across all allopathic orthopaedic surgery programs, demonstrating a statistically significant difference (*P* < 0.001). The highest number of female section chiefs were in pediatrics (14; 9.5%) and hand (12, 10.6%). Oncology had the highest percentage of female faculty represented in section leadership at 18.4%. The lowest number and percentage of female section chiefs was sports medicine (4, 4.8%). When compared to male section chiefs all subspecialties showed a statistically significant difference except for oncology and shoulder and elbow. The breakdown by subspecialty is represented in Table 2.

**Table 2:** Distribution of female orthopaedic surgery section chiefs by Sub-Specialty

Sub Specialty	Total # of Male Section Chiefs	% of Male Faculty	Total # of Female Section Chiefs	% of Female Faculty	*P* value
Total Section Chiefs	808	21.6%	52	10.2%	**< 0.001**
Hand	108	25.9%	12	10.6%	**0.006**
Spine	97	26.7%	2	9.5%	**< 0.001**
Sports Medicine	138	19.1%	4	4.8%	**0.001**
Trauma	122	28.9%	5	10.8%	**< 0.001**
Foot and Ankle	67	26.3%	3	7.5%	**0.009**
Oncology	18	11.3%	7	18.4%	0.231
Pediatrics	92	22.1%	14	9.5%	**< 0.001**
Shoulder and Elbow	29	21.3%	1	9.1%	0.322
Adult Reconstruction	137	22.0%	3	12.5%	**< 0.001**

Evaluation of academic rank demonstrated that 56 (10.9%) female faculty were full professors compared to 622(16.1%) of male full professors (*P* < 0.001). The remaining 457 (89%) female faculty were assistant or associate professors. The subspecialty with the highest percentage of full professors was oncology at 18.4% (7). The lowest was pediatrics at 7.4% (11). Trauma, Pediatrics and adult reconstruction were the subspecialties with a significant difference in the proportion of female full professors when compared to male full professors out of female and male faculty respectively. The breakdown by subspecialty is reported in Table 3.

**Table 3:** Distribution of female orthopaedic surgery full professor faculty by Sub-Specialty

Subspecialty	Total # of Male Full Professors	% of Male Faculty	Total # of Female Full Professors	% of Female Faculty	*P*-Value
Total Full Professors	622	16.1%	56	10.9%	**< 0.001**
Hand	73	14.8%	13	11.5%	0.364
Spine	74	14.5%	2	9.5%	0.834
Sports Medicine	117	16.2%	12	14.5%	0.681
Trauma	97	23.0%	5	10.9%	**0.048**
Foot and Ankle	21	8.3%	3	7.5%	0.869
Oncology	14	8.8%	7	18.4%	0.081
Pediatrics	82	19.7%	11	7.4%	**< 0.001**
Shoulder and Elbow	9	6.62%	1	9.1%	0.710
Adult Reconstruction	135	22.0%	2	8.3%	**< 0.001**

## Discussion

This study explored the distributions of female allopathic orthopaedic surgery faculty members amongst different subspecialties. The analysis demonstrated that adult reconstruction (3.7%), spine (4.8%), and shoulder and elbow (7.4%) had the lowest percentages of female faculty. Additionally, the distribution of female faculty members in section leadership positions and full professorship, was also analyzed showing a lack of representation of female faculty in all subspecialties.

Our findings highlight the need to enhance gender diversity across all orthopaedic subspecialties. We identified those that continue to exhibit low representation, despite the increasing number of female faculty each year. Current literature emphasizes the role of mentorship, provided by both female and male orthopaedic surgeons in the decision of female medical students to pursue a career in orthopaedic surgery [10]. Recent studies evaluating female orthopaedic surgery residents’ fellowship selection reflects these trends. Kavolus et al. identified mentorship and intellectual interest as two primary factors guiding orthopaedic surgery residents in their choice of subspecialty [8]. Additional survey data shows that the reason many female medical students and residents are dissuaded from pursuing specific specialties are due to a lack of feeling of comradery, and work place harassment [15, 19]. Similarly, Liberman et al. highlighted that while male and female residents exhibited similar levels of interest in adult reconstruction, more female residents reported refraining from pursuing this field due to a lack of feeling of belonging, experiences of workplace harassment and lack of encouragement from faculty [9]. Adult reconstruction continues to have the lowest percentage of female faculty at 3.9%, and significant difference in the number of male versus female faculty in full professorship and section leadership.

The proportion of the female faculty that are involved in section leadership stands at 10.2% across all orthopaedic subspecialties and is statistically significantly different than male faculty in section leadership (*P* < 0.001). The data reveals notable disparities among various subspecialties. In the field of sports medicine (4.8%), the proportion of female faculty serving as section chiefs is notably lower. In contrast, oncology had the highest female representation at 18.4%. Oncology having female section chief representation at almost 20% in allopathic academic institutions stands significantly higher than any other subspecialty. Several factors may contribute to the higher representation of females in orthopaedic oncology section leadership. As a relatively small subspecialty, orthopaedic oncology may foster closer mentorship and more supportive professional networks, which have been identified as key influences in specialty selection among female residents, and highly important factors in gaining grant opportunities and consideration for promotion [20]. However, despite being more diverse in section leadership than other subspecialties, a 2023 study on musculoskeletal oncology leadership found that age was the strongest predictor of section leadership, correlating with academic productivity [18]. Academic productivity is an important factor in gaining leadership roles and promotions and a study done by Mulcahey et al., shows that junior female faculty have a significantly lower H index than senior faculty, but that once senior status is achieved the disparities between female and male counterparts research productivity are no longer significant [5].

Trends in demographic characteristics of section chiefs have been investigated in the subspecialty of sports medicine, which has been shown to have one of the lowest percentages of females represented in section leadership. Maqsoodi et al. found that 96% of sports medicine section chiefs were men and identified disparities such as academic productivity and academic rank to be factors influencing this imbalance [11]. Even in subspecialties with higher numbers of female faculty, such as hand and pediatrics, the percentage involved in section leadership roles remains close to the overall percentage of female section chiefs at 10.2%. A recent study also demonstrated a significant decrease in the number females in leadership roles between 2019 and 2020 [13], further demonstrating this discrepancy between females in the field, versus females in leadership roles.

 A similar pattern emerges when considering female faculty with the rank of full professor. The overall percentage of female faculty who are full professors remains low at 10.9%. This trend is consistent across various subspecialties, with the field of pediatrics having the lowest proportion at 7.5%. A study by Shah et al. showed that while the overall percentage of female orthopaedic surgery faculty increased over their study period, that growth within senior faculty positions, which they defined as associate professor or higher, grew at less than half the number of females compared to other medical specialties [ 16 ]. The low number of female full professors in orthopaedic surgery can, in part, be attributed to the historical underrepresentation of females in the field. The pool of eligible female surgeons for promotion to full professor may be limited [ 14 ]. Moreover, factors influencing promotions to full professors, including grant funding, have also been shown to exhibit gender disparities in the literature. Nguyen et al.’s study revealed that, in comparison to their male counterparts, female surgeon scientists were significantly less likely to secure research funding exceeding $750,000 [ 14 ]. These disparities when present throughout many stages of the promotion process slow the rate of growth of female full professors and underscore the importance of addressing gender imbalances throughout academic medicine and promoting equitable opportunities for females in orthopaedic surgery across all subspecialties.

Recommendations and Future Directions.

Recent studies have demonstrated a correlation between the presence of a higher percentage of female faculty in orthopaedic surgery residency programs and an increased number of female residents [7]. Similarly, programs with more female faculty members in leadership positions also have higher numbers of female residents [7]. Diversifying faculty and promoting the inclusion of more female faculty in leadership roles can significantly contribute to recruitment efforts aimed at female residents. However, it is important to acknowledge the complexities associated with the diversification of faculty, particularly in cases where the prevalence of female surgeons within a specific subspecialty remains limited. This inherently results in the number of female surgeons eligible for promotions and leadership positions to be low. Survey data of shows that negative experiences with the operating room or orthopaedic surgery residents and faculty, along with feelings of not “fitting in”, experiences of hazing or concerns about collegiality can limit the number of female identifying medical students from even applying into orthopaedic surgery [5].

**Fig. 1:**
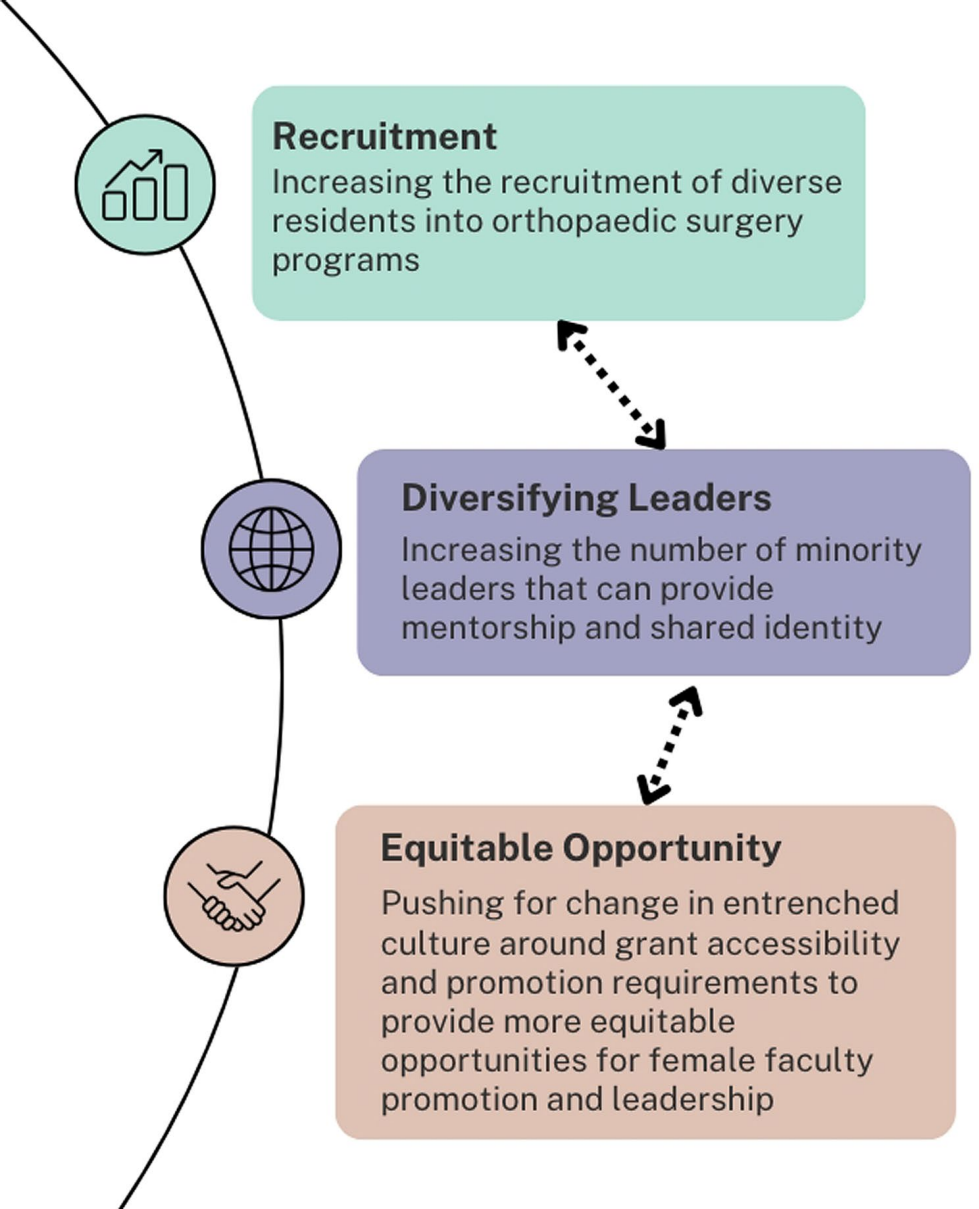
Mentorship, Recruitment and How They Influence Each Other

To effect significant change in the representation of female faulty in section leadership positions and those eligible to be promoted to full professor, active efforts must be taken. Such initiatives include the diversification and advancement of female surgeons within subspecialty committees, fostering research collaborations, and securing grant funding. Both females and males in leadership should actively engage junior female faculty members in various capacities; such as participation in panels, committee work, and in collaborative research endeavors, enhancing their visibility and creating avenues for professional growth. Additionally, data has shown an increase in female junior leadership in the American Society for Surgery of the Hand (ASSH) [12]. Furthermore, survey data underscores the significance of active sponsorship and mentorship for female surgeons, facilitating their career progression within the field [21]. Notably, the value of shared minority identities emerged as an important factor for mentees, further underscoring the importance of diverse mentorship in fostering the advancement of female surgeons [17]. This highlights the importance of not only recruiting more female residents, but also fostering opportunities for female faculty to assume leadership positions, facilitating a more inclusive culture and environment in individual subspecialties, these are highlighted in Fig. 1.

There are several limitations to our study. We used publicly accessible data. This data may not be completely up to date and information may be incomplete. Nine programs listed no information about the gender distribution of their faculty. In addition, faculty were classified as either female or male, which was determined by images and names provided by program websites and pronouns when available. As such, we were limited in our ability to collect data regarding individuals who identify as nonbinary or do not identify within the confines of female versus male. Additionally, designations of leadership positions and academic rank were also determined from what was publicly available on program and hospital websites. Twelve of the program websites had incomplete information on leadership and academic roles, including not listing academic roles on all faculty members, and lack of updates of faculty distinctions. Individual program coordinators were contacted for more detailed demographics of residents and faculty. However, majority of institutions yielded no reply after reaching out two times, leaving data without verification. Future studies may improve on these limitations by sending out surveys to residents and faculty or having multi-institutional studies to aggregate more specific demographic information.

## Conclusion

The overall distribution of female faculty in allopathic orthopaedic academic programs is low at 12.1%. The subspecialties of hand and pediatrics have the highest distribution of female faculty, while oncology has the highest percentage of female faculty in positions of section leadership. Spine and adult reconstruction had the lowest distribution of female faculty in the 2022–2023 academic year. Addressing inequities within academic medicine that are barriers to female orthopaedic surgeons from being considered for promotions, represent foundational measures to enhance diversity within subspecialties and in section leadership. Further research is warranted to identify the factors influencing subspecialty selection and leadership advancement among female residents, to inform actionable efforts that promote gender diversity in Orthopaedic surgery.

## Data Availability

No datasets were generated or analysed during the current study.
